# Supplementary material for: Selection of Ideal Reference Genes for Gene Expression Analysis in COVID-19 and Mucormycosis
Source: Microbiol Spectr. 2022 Nov 15;10(6):e01656-22. doi: 10.1128/spectrum.01656-22 (PMC9769637; doi:10.1128/spectrum.01656-22)
Supplement: Supplemental file 1 — Supplemental material. Download spectrum.01656-22-s0001.pdf, file, 0.4 MB [file spectrum.01656-22-s0001.pdf]

Supplemental Information

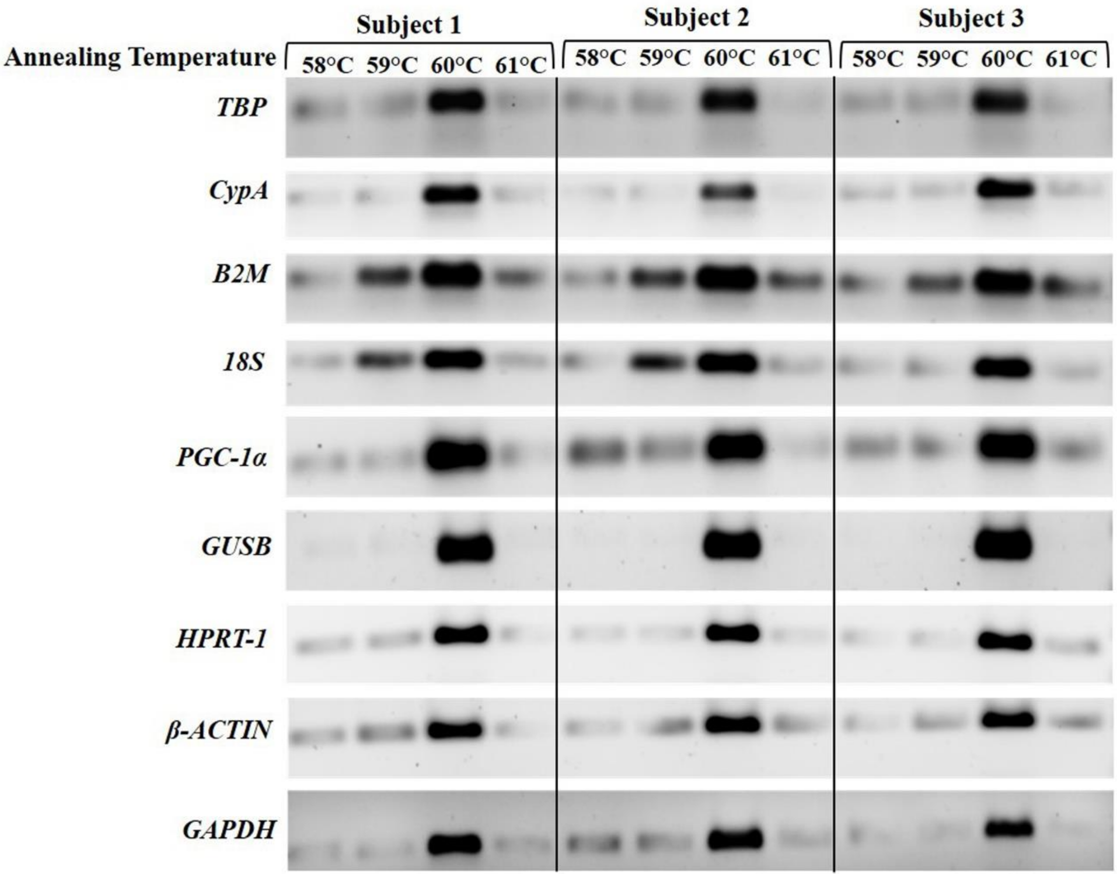

Fig. S1: Determination of an optimum annealing temperature to obtain the maximum amplification efficiency of primers for candidate reference genes. Data show the gene amplicons obtained with their respective primers at different annealing temperature (58°C, 59°C, 60°C, and 61°C). As evident from the data, annealing temperature of 60°C yielded the maximum amplification as highlighted by the band intensity. Subjects 1-3 represent three different healthy individuals; X-axis denotes temperature range used in the gradient-PCR. Left panel (Y-axis) lists the genes used in this study.

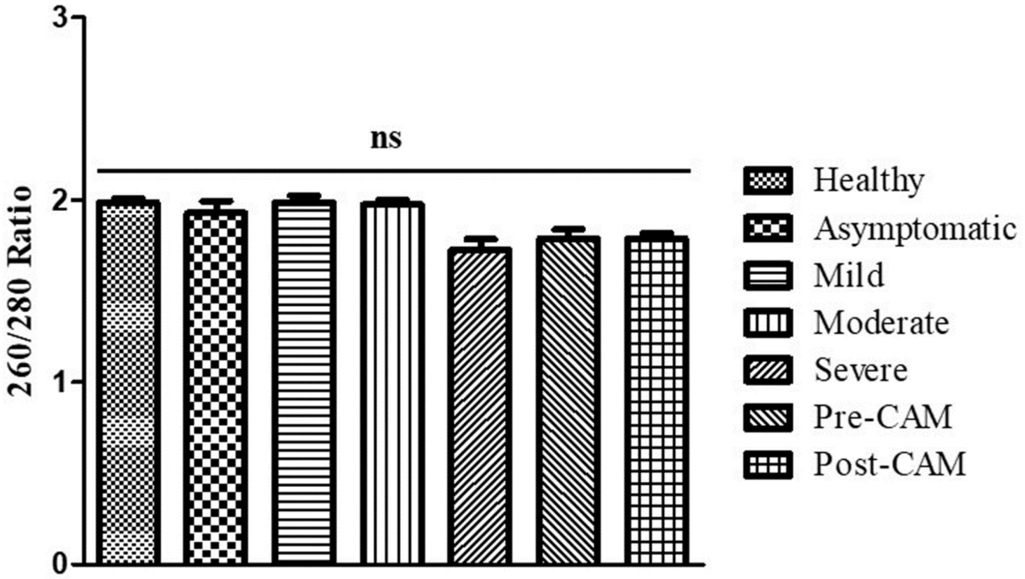

Fig. S2: Mean 260/280 ratio of RNA isolated from various subjects used in the study.

There was no significant difference in the 260/280 ratio across the groups. Data represent mean±SD; One-way ANOVA with Kruskal-Wallis test with Dunn’s post-hoc corrections. “ns” represents non-significant differences (*, *P-value* ≤0.05).

Supplemental Tables

Healthy		Mild		Moderate		Asymptomatic	
Sample Code	*260/280 ratio*	*Sample Code*	*260/280 ratio*	*Sample Code*	*260/280 ratio*	*Sample Code*	*260/280 ratio*
H1	1.938	*A006*	2.021	*B003*	1.900	*D010*	2.010
H2	1.995	*A008*	2.125	*B004*	2.000	*D011*	2.025
H3	1.970	*A010*	2.040	*B011*	1.924	*D012*	2.000
H4	2.001	*A012*	1.900	*B012*	2.013	*D013*	1.980
H5	1.990	*A013*	1.800	*B013*	1.967	*D015*	1.841
H6	2.004	*A027*	1.978	*B010*	2.020	*D017*	1.500
H7	2.100	*A028*	2.046	*B015*	2.100	*D024*	2.041
H8	1.900	*A044*	1.991	*B001*	1.890	*D035*	2.030
**Severe**		**Pre-CAM**		**Post-CAM**			
Sample Code	*260/280 ratio*	*Sample Code*	*260/280 ratio*	*Sample Code*	*260/280 ratio*		
C006	1.929	*CAM03*	1.700	*CAM13*	1.690		
C012	1.622	*CAM8*	1.980	*CAM15*	1.670		
NC001	1.711	*CAM16*	1.735	*CAM34*	1.850		
NC002	1.824	*CAM17*	1.640	*CAM37*	1.810		
NC005	1.725	*CAM19*	1.920	*CAM38*	1.710		
NC008	1.824	*CAM24*	1.800	*CAM4*	1.900		
NC009	1.400	*CAM28*	1.590	*CAM6*	1.826		
NC011	1.792	*CAM47*	1.940	*CAM10*	1.849		

Table S1: Description of RNA quality (260/280) of samples.

Sr. No	Gene	Primer (Forward and Reverse)
1	*TBP* (TATA-box binding protein)	F: 5’ CAGTGACCCAGCAGCATCACT 3’
		R: 5’ AGGCCAAGCCCTGAGCGTAA 3’
2	*CypA* (Cyclophilin)	F: 5’ GTCAACCCCACCGTGTTCTTC 3’
		R: 5’ TTTCTGCTGTCTTTGGGACCTTG 3’
3	*B2M* (β-2-microglobulin)	F: 5’ TGCTGTCTCCATGTTTGATGTATCT 3’
		R: 5’ TCTCTGCTCCCCACCTCTAAGT 3’
4	*18S* (18s ribosomal RNA 5)	F: 5’ CTTAGAGGGACAAGTGGCG 3’
		R: 5’ GGACATCTAAGGGCATCACA 3’
5	*PGC-1α* (PPARG coactivator 1 alpha)	F: 5’ CAGCCTCTTTGCCCAGATCTT 3’
		R: 5’TCACTGCACCACTTGAGTCCAC 3’
6	*GUSB* (glucuronidase beta)	F: 5’ ATGCTGTACCCCCAGGAGA 3’
		R: 5’ TCATTGAAGCTGGAGGGAAC 3’
7	*HPRT-1* (Hypoxanthine phosphoribosyl transferase 1)	F: 5’ GACCAGTCAACAGGGGACAT 3’
		R: 5’ CTTGCGACCTTGACCATCTT 3’
8	*β-ACTIN* (Actin beta)	F: 5’ CGGCATCGTCACCAACTGG 3’
		R: 5’ GCACAGCCTGGATAGCAACGT 3’
9	*GAPDH* (glyceraldehyde-3-phosphate dehydrogenase)	F: 5’ CGAGATCCCTCCAAAATCAA 3’
		R: 5’ TTCACACCCATGACGAACAT 3’
**Candidate gene *NRF2***		
10	*NRF2 (Nuclear factor erythroid 2-related factor 2*)	F: 5’ GAAAATGACAAAAGCCTTCACC 3’
		R: 5’ TTGCCATCTCTTGTTTGCTG 3’
11	IL-6 (Interleukin 6)	F: 5’ AGGAGACTTGCCTGGTGAAA 3’
		R: 5’ ACACACCCACCTTTTTCTGC 3’
12	IL-15 (Interleukin 15)	F: 5’ TGGATGCAAAGAATGTGAGG 3’
		R: 5’ TTGAAATGCCGAGTGTTTTG 3’

Table S2. Primer sequences of candidate reference genes used in this study.
